# Notes and Notices

**Published:** 1890-03

**Authors:** 


### NOTES AND NOTICES.

The Psychology of Epidemics.—Every epidemic carries in its train curious exaggerations of many well-recognized characteristics, and these frequently call for appreciation and for treatment almost as much as the disease in which they originate. Perhaps one of the most striking of these mental perversities is to be found in the idea that the epidemic is to be treated by "common sense," or by nostra which have been largely advertised, or by specifics which are known to the laity mainly through their frequent mention in the daily press. Those suffering under this delusion feel that it is wholly unnecessary to seek skilled assistance, and they boldly dose themselves with remedies of whose power and properties they are absolutely ignorant. In Vienna it has already been found necessary to forbid the sale of antipyrine, except under doctors' prescriptions, as no less than seventeen deaths were attributed to stoppage of the heart's action owing to overdoses. The freedom with which the prescription of this remedy has been assumed by the public has long since been viewed with anxiety by the medical profession, and frequent warnings have already fallen upon deaf ears; and yet it is to be feared that if the epidemic of influenza should spread, many more examples of recklessness will have to be recorded. Mr. Labouchere, claiming to act "by the light of common sense," upon having "a cough, a headache and an all-overish ache," "accompanied by sneezing," diagnosed the prevailing epidemic, and at once administered to himself "thirty grains of quinine," and to meet the cough he took "unlimited squill pills." He writes that the one "settled the fever" and the other "settled the cough," and that in four days he was quite well. Upon this last fact he is certainly to be congratulated, though we trust that others may not be impelled, "by the light of common sense," to follow him in such heroic measures or to emulate his example by trying the effect of antipyrine in similar unlimited doses. It is serious enough to cope with an epidemic and its sequelæ without having matters complicated by ignorant and reckless experimental therapeutics.—*London Lancet.*

Dr. W. B. Clarke, Secretary of the Indiana Institute of Homœopathy, was married to Miss Alice P. Winings on Tuesday evening, February 4th, at the home of the bride, 188 Blackford Street, Indianapolis, by the Rev. F. A. Guthrie. The house was filled with enthusiastic friends who came to wish good luck to the happy couple.

A Snapping-Turtle Baby.—A child resembling in many respects both a catfish and a snapping-turtle was born of colored parents in the upper part of Trenton, New Jersey, on Friday night, January 31st. The weight of the child is about seven pounds and the head and trunk of the body are perfectly natural in form, but there are neither arms nor legs. It has an abundance of black, curly hair, which completely covers the forehead, from the eyebrows. The bridge of the nose begins between the eyes and is about a half-inch in length. At its point there are two holes, one on either side, and a partly-formed mouth. Beneath this partly-formed mouth is another small projection similar to that of the end of the nose, and beneath it, where the mouth should be, is a wide gash, but no upper lip.

The chin is perfect and there are two perfectly formed ears, but they are entirely closed. On either side of the body, in the vicinity of the shoulders, is a projection similar in shape to the claw of a snapping-turtle or the fin of a catfish, and on either side of the lower portion of the trunk, just below the hips, are similar fin-like projections. The parents of the child are educated colored people, and the attending physician, Dr. A. H. Dey, is unable to account for the monstrosity. The child was dead when born and the body will be presented to the medical college of the University of Pennsylvania.

M. Pasteur.—Says a Paris correspondent: "Paralysis, if I am not much mistaken, is stealing a quick march on M. Pasteur. He had one attack some years ago which left him with a dead leg. The eyelids are now all but inert, and I noticed that the timbre of his voice had altered for the worse, and that the speech was thick and embarrassed. There were wild twitches in his face, but his mind was as clear as ever."

Treatment of Stammering.—"It is said that stammerers rarely if ever show any impediment to speech when speaking in whispers. On this fact a new method of treatment has been advocated by Dr. Coen, which is as follows: In the first ten days speaking is prohibited. This will allow rest to the voice, and constitutes the preliminary state of treatment. During the next ten days speaking is permissible in the whispering voice, and in the course of the next fifteen days the ordinary conversational tone may be gradually employed."—*Druggists' Circular and Chemical Gazette.*

